# Clinical and Electrocardiographic Evaluation of Sickle-Cell Anaemia Patients with Pulmonary Hypertension

**DOI:** 10.5402/2012/768718

**Published:** 2012-03-25

**Authors:** N. I. Oguanobi, E. C. Ejim, B. C. Anisiuba, B. J. C. Onwubere, S. O. Ike, O. G. Ibegbulam, O. Agwu

**Affiliations:** ^1^Department of Medicine, University of Nigeria Teaching Hospital, Enugu, Nigeria; ^2^Department of Medicine, Federal Medical Centre Asaba, Delta State, Nigeria; ^3^Department of Haematology, University of Nigeria Teaching Hospital, Enugu, Nigeria

## Abstract

Pulmonary hypertension is an emerging complication of sickle cell anaemia with associated increased risk of mortality. In order to evaluate the clinical and electrocardiographic findings in adult sickle-cell patients with pulmonary hypertension, a cross sectional study was conducted on sixty two sickle cell anaemia patients and sixty two age and sex matched normal controls. Elevated pulmonary artery pressures (PAP), defined by PAP ≥ 30 mm Hg on echocardiography, was demonstrated in 41.9% of patients with sickle cell anaemia and in 3.2% of the controls; *χ* ^2^ = 26.571, *P* < 0.001. Right ventricular hypertrophy, increased P-wave duration, QTc interval, and QTc dispersion were significantly associated with pulmonary hypertension. Significant correlation was found between mean PAP and (1) Frequency of crisis (Spearman correlation = 0.320; *P* = 0.011), (2) body mass index (Pearson's correlation = −0.297; *P* = 0.019), and (3) QTc interval (Pearson's correlation 0.261; *P* = 0.040). Pulmonary hypertension in adult sickle anaemia patients is associated with electrocardiographic evidence of right ventricular hypertrophy, and correlates significantly with frequency of vaso-occlusive crisis, and QTc interval. The observations by this study tend to suggest that these parameters could be useful for early detection and prevention of pulmonary hypertension in patients with sickle cell anaemia.

## 1. Introduction

 Pulmonary hypertension is an increasingly recognized complication of sickle-cell anaemia and a risk factor for early death [1–4]. Recurrent episodes of acute and subacute pulmonary crises reflecting in situ sickling within the lung has been postulated to be the primary event leading to pulmonary hypertension. Haemolysis may participate in its pathogenesis by limiting nitric oxide (NO) bioavailability and producing vasculopathy [5, 6]. 

An initial study in Howard University, USA, using echocardiographic assessment of tricuspid valve regurgitant jet velocity ≥2.5 m/sec as diagnostic criteria, demonstrated pulmonary hypertension in 32% of adult sickle-cell patients, and the prevalence appeared to increase with age of the patients [7]. In patients between 40 and 49 years old, the prevalence was 40% and increased to 55–60% by age 50 and above. Other studies have documented prevalence rates between 20 and 40% [4, 8–10].

Sickle-cell anaemia patients with pulmonary hypertension have a significantly increased mortality rate compared with patients without pulmonary hypertension. Sutton and colleagues [4] reported 40% mortality rate in sickle-cell patients with pulmonary hypertension at 22 months after diagnosis (odd ratio 7.86; 95% confidence interval = 2.63–23.4) compared with sickle-cell patients without pulmonary hypertension. Castro et al. [11] in a study of 34 adult sickle-cell patients who underwent right heart catheterization for evaluation of pulmonary hypertension found increased pulmonary artery pressure in 20 (58.8%) on initial catheterization. During 23–45 months of follow up, 11 of these 20 (55.0%) died compared with 3 of the 14 without pulmonary hypertension (21.0%). Every 10 mmHg increase in mean pulmonary artery pressure was associated with 1.7 increase in mortality (95% confidence interval = 1.1–2.7; Cox proportional hazard model, *P* = 0.028). Little is known about electrocardiographic findings in sickle-cell patients with pulmonary hypertension. The study was aimed at comparing the clinical and electrocardiographic findings in sickle-cell anaemia patients with raised pulmonary artery pressure with those of patients without pulmonary hypertension. 

## 2. Methodology

### 2.1. Patients/Participants

The study was carried out in the adult outpatient sickle-cell clinic and the Cardiac Centre of the University of Nigeria Teaching Hospital (UNTH), Enugu, Nigeria. The study subjects were drawn from adult patients (age ≥ 18 years) [12], attending the adult sickle-cell clinic of the hospital, who had haemoglobin genotype SS on haemoglobin electrophoresis, were in steady state, and consented to participate in the study. Steady state is defined as absence of any crisis in the preceding four weeks, absence of any symptoms, or signs attributable to acute illness. A total of sixty two sickle-cell anaemia patients, and sixty two age- and sex-matched normal controls were studied. 

### 2.2. Study Procedure

All the subjects had clinical evaluation, electrocardiographic and echocardiographic examinations. Resting 12-lead electrocardiography was performed on all subjects using cardioline Ar-600 model electrocardiography machine at a paper speed of 25 mm/s and standardized at 0.1 mv/mm. A single observer analyzed the electrocardiogram. Measurements of the heart rate, cardiac axis, PR- interval, QRS duration, and QTc interval were done in the standard fashion. Heart rate correction of the QT interval was performed using Bazett's formula (QTc = QT/√ RR) [13]. The dispersion of P-wave, QRS, and QTc intervals was measured manually under magnifying glass by the same observer and was taken as the difference between the maximum and minimum values of each parameter on standard 12-lead electrocardiogram. Left ventricular hypertrophy on electrocardiogram was based on Sokolow and Lyon voltage criteria [14], while right ventricular hypertrophy was based on the criteria described by Allenstein and Mori (dominant or tall R-waves or Rs pattern in a VR, V_1_, and V_2_ with deep S-wave in I, aVL, V_5_, and V_6_) [15]. 

Echocardiography was done using Hewlett Packard Sonos 2500 echocardiography machine with 3.7 MHz transducer. Pulmonary artery flow acceleration time was obtained from a Doppler signal of the pulmonary flow in the parasternal short-axis view at the aortic valve level and was described as the time from onset to peak flow velocity [16]. The mean pulmonary artery pressure (mean PAP) was calculated from the formula: [16] mean PAP (**m** **m**/**H** **g**) = 90 − (0.62 × **A** **T**), where AT is the acceleration time of the pulmonary artery flow. Pulmonary hypertension was defined as calculated mean pulmonary artery pressure ≥ 30 mm/Hg [4, 7].

### 2.3. Ethical Consideration

Ethical clearance for the study was obtained from the Ethical Committee of the University of Nigeria Teaching Hospital, Enugu, Nigeria. All the participants were treated in accordance with the requirements of good clinical practice. The Declaration of Helsinki's recommendations for guiding physicians in biomedical research involving human subjects were followed [17].

### 2.4. Data Analysis

Data were presented as means ± standard deviation for continuous variables and as proportions for categorical variables. Comparisons of continuous variables between groups were made with independent Student's *t*-test. For discrete variables, distribution between groups was compared with chi-square test and Fishers exact test as appropriate (where an expected cell is less than 5). Multivariate Pearson's correlation coefficient was used to determine the relations between clinical, electrocardiographic data and mean pulmonary artery pressure. All statistical analyses were carried out using the Statistical Packages for Social Sciences (SPSS Inc., Chicago, Illinois) software version 11.0 and EPi-Info version 3.4. Statistical tests with 2-tailed probability values less than 0.05 were considered statistically significant.

## 3. Results

### 3.1. Baseline Clinical Characteristics

 A total of 62 patients and 62 control subjects were studied. The age, gender, and anthropometric parameters of the patients and controls are shown in Tables 1 and 2.

### 3.2. Mean Pulmonary Artery Pressure

 Values for pulmonary artery pressure in the patients and controls are shown in Table 3. Mean pulmonary artery pressure was significantly higher in patients compared with controls. No significant age and gender difference in pulmonary artery pressure was observed. 

### 3.3. Prevalence of Pulmonary Hypertension

**Figure 1:**
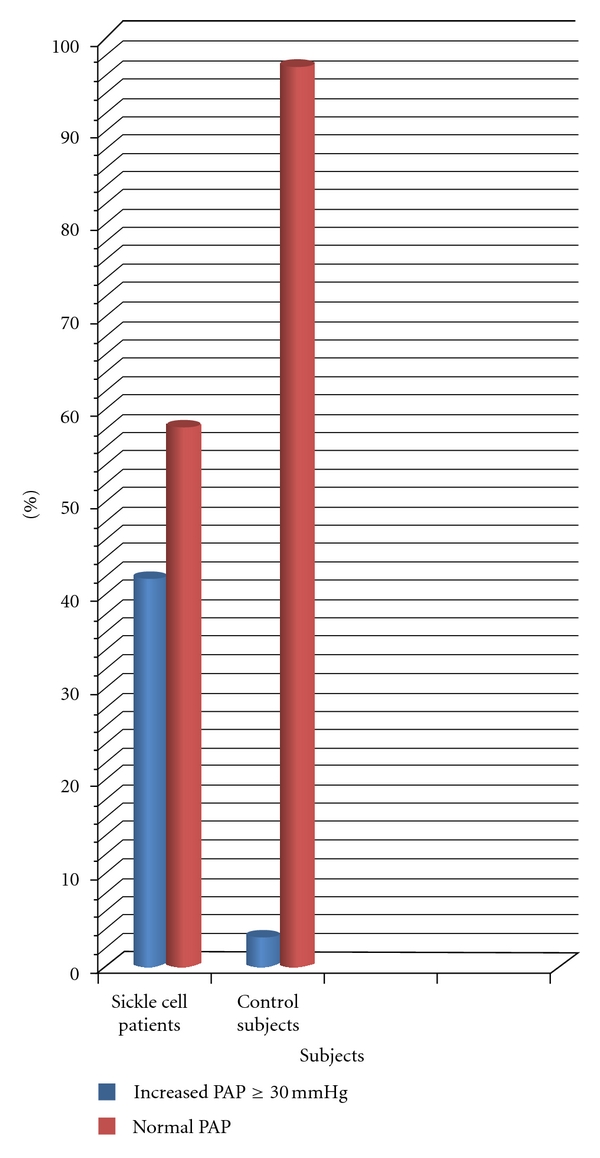
Prevalence of increased pulmonary artery pressure in patients and contols.

 Elevated pulmonary artery pressures (PAP) as defined by PAP ≥ 30 mmHg was demonstrated in (26) 41.9% of patients with sickle-cell anaemia and in (2) 3.2% of the controls; *χ* ^2^ = 26.571, df = 1, *P* < 0.001 (Figure 1).

### 3.4. Relationship between Clinical and ECG Parameters and Pulmonary Hypertension

Right ventricular hypertrophy was significantly associated with pulmonary hypertension (Table 4). The mean frequency of crisis per year was higher in patients with pulmonary hypertension. History of recurrent acute chest syndrome was noted in 75.9% of the patients. P-wave duration, QTc interval, and QTc dispersion were increased in patients with pulmonary hypertension (Table 5). 

Significant correlation was found between mean PAP and (1) frequency of crisis (Spearman correlation = 0.320; *P* = 0.011), (2) body mass index (Pearson's correlation = −0.297; *P* = 0.019), and (3) QTc interval (Pearson's correlation 0.261; *P* = 0.040), (Table 6). 

## 4. Discussion

The prevalence of pulmonary hypertension in adult Nigerian sickle-cell anaemia patients in this study was 41.9%. This value is slightly higher than previous report of 25% by Aliyu et al. [10] in Northern Nigeria. However, in that study, neither the mean PAP nor the mean age of the subjects was stated. 

Previous studies have relied on tricuspid regurgitant jet velocity as an indirect estimate of PAP. This has resulted in an underestimation of the prevalence of pulmonary hypertension in sickle-cell anaemia patients. Results of right heart catheterization for evaluation of pulmonary artery pressures in sickle-cell patients have shown prevalence rate of 58.8% [11]. Autopsy studies suggest that up to 75% of sickle-cell anaemia patients have histological evidence of pulmonary arterial hypertension at the time of death [18]. Mean PAP derived from the echocardiographic estimation of pulmonary artery flow acceleration time in steady-state patients as was used in this study has been shown to correlate significantly with values from cardiac catheterization [16].

 Several studies have corroborated the role of vaso-occlusive crisis and acute chest syndrome in initiating nitric oxide depletion resulting in the pathological changes in pulmonary hypertension [6, 19]. This study identified a positive correlation between pulmonary artery pressure and frequency of vaso-occlusive crisis.

 Patients with pulmonary hypertension were found to have reduced body mass index when compared with patients without pulmonary hypertension. This difference could be due to increased disease severity in patients with pulmonary hypertension.

 Recurrent insitu vaso-occlusive crisis in the pulmonary vascular bed with resultant pulmonary hypertension and right ventricular hypertrophy has been demonstrated in sickle-cell disease patients [20]. This is in keeping with the finding by this study of a significantly increased prevalence of right ventricular hypertrophy in patients with pulmonary hypertension. Right ventricular hypertrophy in these patients explains the significant positive correlation between pulmonary artery pressure and QTc interval. The significance of increased P-wave duration and the spatial dispersion of QTc-interval noted in this study in patients with pulmonary hypertension are unclear. QTc dispersion is a measure of the disparity among QTc intervals in various electrocardiographic leads and reflects the variability of myocardial repolarization [21]. This corroborates the finding in a study by Akgul et al. in Turkey which recorded higher QTc dispersion in sickle-cell patients with pulmonary hypertension [22].

## 5. Conclusion

 Pulmonary hypertension in adult sickle-cell anaemia patients is significantly associated with electrocardiographic evidence of right ventricular hypertrophy, increased P-wave duration, QTc interval, and QTc dispersion and correlates significantly with frequency of vaso-occlusive crisis and QTc interval. The observations by this study tend to suggest that these parameters could be useful for early detection and prevention of pulmonary hypertension in patients with sickle-cell anaemia.

## Figures and Tables

**Table 1:** Age, gender, and anthropometric data.

parameters	SCA	Controls	*t*-test	*P* value
	mean (SD)	mean (SD)		
Age (years)	28.27 (5.58)	28.37 (5.91)	0.987	0.924
Gender {frequency (%)}				
Male	31 (50)	31 (50)	0.000	1.00^a^
Female	31 (50)	31 (50)		

Total	62	62		
Weight (kg)	54.97 (10.61)	67.35 (8.37)	7.20	<0.001*
Height (m)	1.62 (0.14)	1.72 (0.07)	4.960	<0.001*
Body surface area (m^2^)	1.62 (0.03)	1.78 (0.14)	3.723	<0.001*
Body mass index (Kg/m^2^)	20.47 (2.73)	23.87 (3.22)	6.181	<0.001*

* Statistically significant (2 sided); ^a^chi-square.

SCA—sickle-cell anaemia.

**Table 2:** Age distribution of patients and controls.

Age range	Frequency		*χ* ^2^	df	*P* value
	Sickle-cell patients	Controls			
18–22	26	27			
23–27	21	20			
28–32	10	10			
33–44	5	5			

Total	62	62	0.043	3	0.999

**Table 3:** Mean pulmonary artery pressure in patients compared with controls.

Age range	Mean PAP ± SD		*t*-test	df	*P* value
	Sickle-cell patients	Controls			
18–22	19.18 (9.79)	10.38 (6.99)	3.408	51	0.001*
23–27	24.42 (6.68)	7.09 (6.02)	5.325	28	0.001*
28–32	23.09 (7.07)	7.86 (4.61)	3.758	18	0.001*
33–44	23.05 (4.96)	10.09 (6.25)	1.121	8	0.295

Total	21.80 (8.77)	8.60 (2.99)	6.785	118	0.001*

* Statistically significant (2 sided), PAP = pulmonary artery pressure, and SD = standard deviation.

**Table 4:** Clinical and electrocardiographic parameters; association with pulmonary hypertension.

Parameters	Frequency (%)		*χ* ^2^	*P* value
	Increased PAP (*n* = 26)	Normal PAP (*n* = 36)		
Male gender	12 (46.15)	19 (52.78)	0.265	0.797
Female gender	14 (53.85)	17 (47.22)		
Loud P2	23 (88.46)	29 (80.56)		0.4985^a^
LVH	12 (46.15)	32 (88.89)	13.382	<0.001*
LAE	9 (34.62)	14 (38.89)	0.118	0.794
RVH	13 (50.00)	5 (13.89)	7.883	0.005*

* Statistically significant (2 sided), ^a^ Fisher's exact test, P2 = pulmonary component of second heart sound, LVH = left ventricular hypertrophy, LAE = left atrial enlargement, and RVH = right ventricular hypertrophy.

**Table 5:** Comparison of clinical and electrocardiographic parameters in patients with normal and increased pulmonary pressure.

Parameters	Values (mean SD)		*t*- test	*P* value
	Increased PAP	Normal PAP		
Age	25.08 (5.96)	23.64 (6.29)	1.011	0.316
Pulse rate	86.42 (6.99)	88 (5.19)	1.052	0.291
BMI	19.91 (2.11)	21.42 (3.23)	2.215	0.031*
Mean crisis frequency/year	3.20 (1.52)	2.22 (1.19)	2.703	0.009*
Systolic BP	121.00 (11.83)	119.28 (12.17)	0.556	0.580
Diastolic BP	63.00 (10.04)	66.50 (8.06)	1.521	0.133
MAP	79.12 (7.71)	83.26 (6.77)	1.284	0.204
Haematocrit	24.96 (2.73)	23.65 (3.34)	1.643	0.106
P-wave duration	152.3 (21.42)	109.4 (14.72)	9.354	0.0001*
P-wave dispersion	62.3 (19.74)	64.4 (17.31)	0.460	0.647
PR-interval	197.7 (28.5)	196.4 (32.08)	0.165	0.869
QRS duration	88.5 (19.74)	87.2 (18.61)	0.252	0.802
QRS dispersion	52.3 (7.22)	52.2 (6.62)	0.016	0.987
QTc interval	433.8 (38.69)	398.6 (18.61)	4.760	0.0001*
QTc dispersion	105.0 (23.24)	90.8 (18.54)	2.675	0.0096*

* Statistically significant (2 sided), BMI = body mass index, and MAP = mean arterial blood pressure.

**Table 6:** Pulmonary artery pressure; correlation with some clinical and electrocardiographic parameters.

Parameters	Patients		Controls	
	*r*	*P* value	*r*	*P* value

Age	0.149	0.249	−0.003	0.983
Freq. of crisis	0.308^∗a^	0.015	—	—
BMI	−0.297*	0.019	0.226	0.077
BSA	−0.176	0.170	0.126	0.329
MAP	−0.103	0.423	−0.156	0.225
Haematocrit	0.171	0.185	0.230	0.072
Heart rate	0.093	0.474	0.112	0.387
QTc	0.262*	0.040	0.250	0.050

* Statistically significant (2 sided), *r* = Pearson's correlation coefficient, *P* = probability value, ^a^ Spearman correlation, BMI = body mass index, BSA = body surface area, and MAP = mean arterial blood pressure.
